# Esophageal fistula: serious complication after endoscopic thyroidectomy : A case report and literature review

**DOI:** 10.1097/MD.0000000000044216

**Published:** 2025-08-29

**Authors:** Jiamin Xu, Ying Zhou, Juan Lin, Huihong He

**Affiliations:** a Department of Breast and Thyroid Surgery, Shaoxing People’s Hospital, The First Hospital of Shaoxing University, Shaoxing, Zhejiang Province, China.

**Keywords:** endoscopic thyroidectomy, esophageal fistula, surgical nursing, thyroid cancer

## Abstract

**Rationale:**

Esophageal fistula is a rare and serious complication after endoscopic thyroidectomy. Current research focuses more on surgical treatment, while there are few treatment plans for esophageal fistula after endoscopic thyroidectomy. There is currently no standardized intervention for esophageal fistula after thyroid surgery.

**Patient concerns:**

The patient wants to cure the esophageal fistula as soon as possible and does not want to have other complications.

**Diagnoses:**

The patient was diagnosed with thyroid cancer.

**Interventions:**

We summarize the key points of interventions as follows: First, a comprehensive assessment of the patient’s condition was conducted before the surgery, and an individualized surgical approach was formulated. During the operation, preventive intervention measures were carried out. Second, after the surgery, fasting, gastrointestinal decompression, and supportive treatment were provided. Then, an individualized follow-up plan was developed after discharge to prevent long-term complications. At the same time, the negative emotions of the patients were also paid attention to, and timely counseling was given to help them return to society as soon as possible.

**Outcomes:**

The patient was hospitalized for 35 days. After the joint efforts of doctors, nurses, and patients, she recovered well and was discharged from the hospital 31 days after surgery. Two-month follow-up after discharge showed that gastrointestinal function recovered well, with no abdominal pain, bloating, diarrhea, nausea, vomiting, and other discomforts, and no long-term complications such as esophageal stenosis.

**Lessons:**

The interventions we summarized are effective and feasible for patients with esophageal fistula after endoscopic thyroidectomy.

## 1. Introduction

Thyroid cancer is the most common malignant tumor of the endocrine system in women.^[1]^ According to the 2022 global cancer statistics, the number of new cases is 8,21,000, and the incidence rate is continuing to grow.^[2]^ Surgery is the cornerstone of thyroid cancer treatment. In recent years, endoscopic thyroidectomy has become increasingly popular in clinical applications due to its advantages such as minimal trauma, microincision, and safety and feasibility.^[3]^ However, due to the location of tumor growth, surgery may cause esophageal damage and esophageal fistula. This is a rare and serious complication in clinical practice, and there are currently few nursing reports on esophageal fistula after endoscopic thyroidectomy.^[4,5]^ In this case report, we described a patient with esophageal fistula after endoscopic thyroidectomy for thyroid cancer. By establishing a multidisciplinary collaborative team and working together with doctors, nurses and patients, the patient recovered and was discharged smoothly, and no obvious complications were found in the routine postoperative follow-up.

## 2. Case presentation

**Figure 1.**
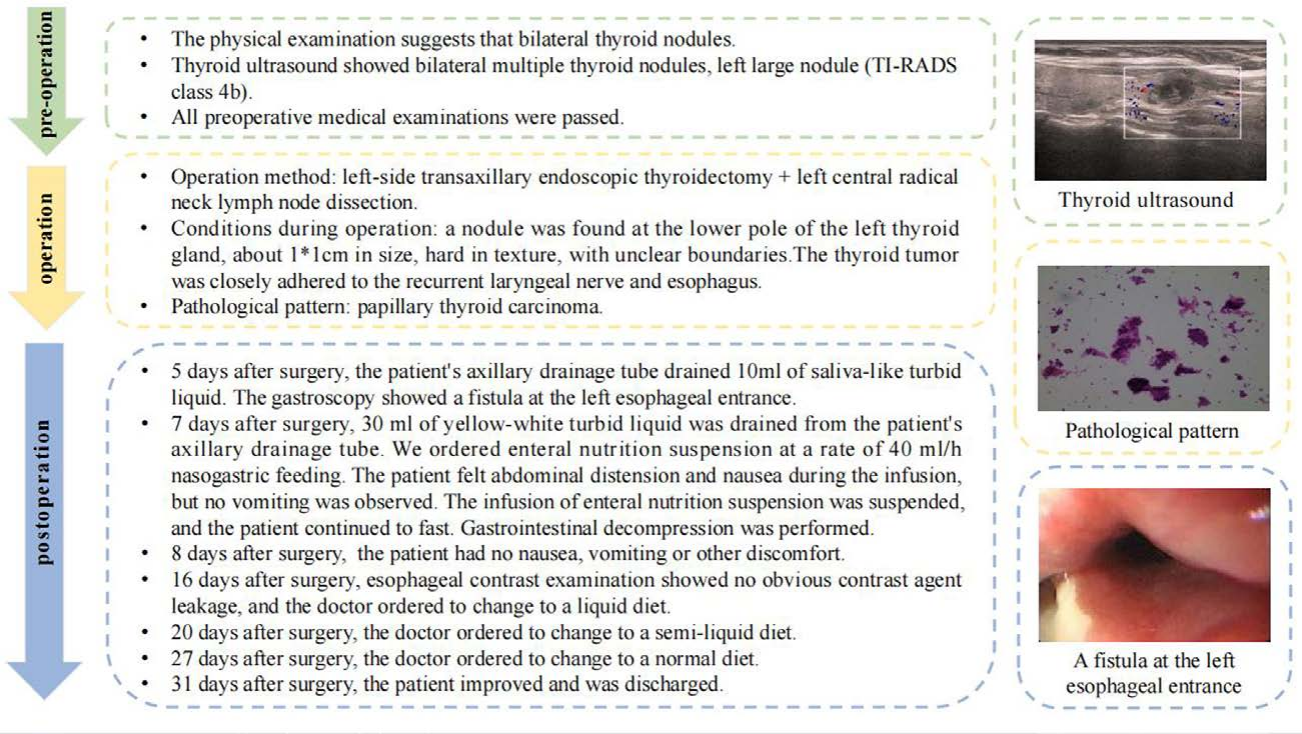
As shown in the figure, the whole course of the disease is described from 3 parts: pre-operation, intra-operation and post-operation. The pictures on the right are representative clinical data of patients in these 3 stages. The first picture is the B-ultrasound of the patient’s thyroid gland, the second picture is the pathological report of the patient, and the third picture is the esophageal fistula of the patient seen under the endoscope. TI-RADS = Thyroid Imaging Reporting and Data System.

As shown in Figure 1, the 22-years-old female was admitted to the hospital due to thyroid nodules in March, 2024. The physical examination suggests that bilateral thyroid nodules. Thyroid ultrasound showed bilateral multiple thyroid nodules, left large nodule (Thyroid Imaging Reporting and Data System class 4b). The patient had undergone a biopsy before the operation, and the pathological result showed that it was papillary thyroid carcinoma. Through preoperative conversations with this patient, we fully respected her wishes and formulated an individualized surgical plan. The patient underwent left-side transaxillary endoscopic thyroidectomy + left central radical neck lymph node dissection. During the operation, a nodule was found at the lower pole of the left thyroid gland, about 1 × 1 cm in size, hard in texture, with unclear boundaries. The thyroid tumor was closely adhered to the recurrent laryngeal nerve and esophagus. The pathological results showed papillary thyroid carcinoma. All lymph nodes were negative and there was no tumor cell invasion. The patient’s vital signs (VS) were stable after the operation, and she was given symptomatic and supportive treatments such as fasting, gastrointestinal decompression, antiemetic, stomach protection, anti-inflammatory, nutrition and fluid supplementation according to the doctor’s advice. The patient did not experience hoarseness or numbness of hands and feet after the operation. However, 5 days after surgery, the patient’s axillary drainage tube drained 10 mL of saliva-like turbid liquid, which was considered to be a possible esophageal suture leakage. A gastroscopy was performed. Based on the results of gastroscopy, we found a fistula at the left esophageal entrance. The patient’s gastric tube was kept in place according to the doctor’s advice. The radiology examination confirmed that the gastric tube was in the stomach. Seven days after surgery, 30 mL of yellow–white turbid liquid was drained from the patient’s axillary drainage tube. The doctor ordered 500 mL of enteral nutrition suspension at a rate of 40 mL/h nasogastric feeding. The patient felt abdominal distension and nausea during the infusion, but no vomiting was observed. The infusion of enteral nutrition suspension was suspended, and the patient continued to fast. Gastrointestinal decompression was performed, and 170 mL of light yellow turbid gastric contents were drained. Eight days after surgery, the doctor ordered to continue enteral nutrition support, and the patient had no nausea, vomiting or other discomfort. Sixteen days after surgery, esophageal contrast examination showed no obvious contrast agent leakage, and the doctor ordered to change to a liquid diet. Twenty days after surgery, the doctor ordered to change to a semiliquid diet. Twenty seven days after surgery, the doctor ordered to change to a normal diet. Thirty one days after surgery, the patient improved and was discharged. Follow-up 2 months after discharge showed that the patient was in good general condition and no long-term complications such as esophageal stenosis occurred.

## 3. Discussion

Several studies have reported esophageal perforation following thyroid surgery. Irawan et al reported^[6]^ that A 43-year-old female was diagnosed with esophageal perforation following thyroidectomy 2 weeks later. A delay in diagnosis and treatment for esophageal perforation leads to severe complications, highlighting the need for standardized treatment algorithms. Over a 3-week period, serial imaging demonstrated fistula closure and complete wound healing by 8 weeks. They pointed out that the involvement of a multidisciplinary team from the very first identification of surgical complications is crucial for ensuring proper treatment. This is consistent with our key focus. Ward et al reported^[7]^ that a 56-year-old female developed neck pain and swelling 3 days after thyroid lobectomy operation. There was a thick, yellowish exudate adherent to the involved surfaces, and the strap muscles were noted to be thickened, stiff and friable. The patient demonstrated a esophageal leak. Because of variability in clinical presentation, however, early diagnosis is often difficult, which may lead to treatment delays. Esophageal perforation is a life-threatening complication with a high mortality rate, and must be promptly diagnosed and appropriately managed to prevent further complications and death.

### 3.1. Early identification of postoperative complications and proactive treatment of the causes

Esophageal fistula is a serious short-term complication after laparoscopic thyroid surgery. It is rare in clinical practice. Literature reports that the incidence of esophageal fistula after thyroid surgery is 0.2% to 4.2%.^[7]^ The main manifestations of esophageal fistula are saliva or other fluids flowing out of the surgical wound during surgery, or increased body temperature of the patient after surgery, local redness and swelling of the wound, and saliva-like or turbid fluid flowing out of the drainage tube.^[8–10]^ In this case, according to the preoperative imaging examination and intraoperative situation, the patient’s thyroid tumor was closely adhered to the recurrent laryngeal nerve and esophagus, and the possibility of postoperative esophageal fistula was high. The patient returned to the ward safely after surgery, and the responsible nurse closely observed her VS, wounds and axillary drainage fluid, paying attention to whether there was a complication of esophageal fistula. One to four days after surgery, the patient’s VS were stable, the wound was locally dry without redness and swelling, and the axillary drainage tube drained a pale blood-colored clear liquid. Five days after surgery, the patient’s axillary drainage tube drained 10 mL of saliva-like turbid liquid, which was immediately reported to the doctor. After endoscopic examination confirmed that it was an esophageal fistula, the cause was immediately treated. First, fasting was adopted, enteral nutrition support was provided through a nasogastric tube, and the wound negative pressure drainage bottle was replaced with a negative pressure drainage ball. The ball was squeezed every shift to ensure adequate drainage of the wound, and anti-inflammatory symptomatic treatment was strengthened. After the above conservative treatment, 16 days after surgery, no obvious contrast agent leakage was found in the esophageal angiography, indicating that the esophageal fistula had healed on its own. If the esophageal fistula is large or conservative treatment fails, another surgery is required. In addition, surgeons should conduct a thorough and comprehensive assessment before the operation, especially the anatomical positions of the thyroid gland, trachea and esophagus. Then, an individualized surgical plan should be formulated. It is recommended to perform a multidisciplinary joint operation to minimize surgical risks and reduce the incidence of postoperative complications.

### 3.2. Implement gradual nutritional management to maintain homeostasis

Nutritional management is an important part of the comprehensive treatment of patients with esophageal fistula.^[11,12]^ Many guidelines proposed that nutritional screening and evaluation should be performed again after surgery.^[13,14]^ The nutritional plan should be adjusted according to the patient’s gastrointestinal function and individual tolerance, and the patient’s tolerance should be closely evaluated to avoid the occurrence of adverse events such as intestinal ischemia caused by excessive intestinal burden.^[12,13]^ The patient’s preoperative nutritional risk screening 2002 score was 1 point, body mass index (BMI) was 24.11 kg/m^2^, serum total protein was 71.3 g/L, albumin was 45.8 g/L, there was no electrolyte disorder, and the nutritional status was good. After surgery, the NRS 2002 score was 4 points, BMI was 22.46 kg/m^2^, serum total protein was 63.6 g/L, albumin was 39.5 g/L, electrolyte indicators were normal, there was a nutritional risk, and an individualized nutritional management plan was formulated after consultation with the nutrition department. Nutritional support follows the principle of “from less to more, step by step.”^[15]^ Seven days after surgery, the doctor ordered 500 mL of enteral nutrition suspension to be fed through nasogastric tube at a rate of 40 mL/h, and the infusion rate of enteral nutrition emulsion and infusion volume was gradually increased. The temperature of enteral nutrition solution was maintained at 38°C to 40°C, and the pipeline was flushed with 20 mL of warm water every 4 hours to keep the pipeline unobstructed. On the same day, the patient felt abdominal distension and nausea during the infusion of enteral nutrition emulsion, but no vomiting. Considering the reasons such as surgical stress and fasting, the patient had mild gastroparesis symptoms. In order to avoid the reflux of nutrition solution and gastric juice to corrode the fistula, the doctor ordered to suspend the infusion of enteral nutrition emulsion and the patient was keeping fasting, gastrointestinal decompression, drain 110 mL of light yellow turbid gastric contents, and recommended the patient to get out of bed more to promote the recovery of gastrointestinal digestion and absorption function. Then, the doctor ordered to continue enteral nutrition support on the second day, and the patient had no nausea, vomiting and other discomfort. Sixteen days after surgery, esophageal contrast examination was performed, and no obvious contrast agent leakage was found. The doctor ordered to change to a liquid diet. Twenty days after surgery, the doctor advised to change to a semiliquid diet. Twenty seven days after surgery, the doctor advised to change to a normal diet. Before discharge, the patient’s BMI was 22.1 kg/m^2^, serum total protein was 66.8 g/L, albumin was 40.8 g/L, there was no electrolyte disorder, and the nutritional status was good.

### 3.3. Develop individualized follow-up treatment plans to prevent long-term complications

The main long-term complication after thyroidectomy for esophageal invasion is secondary esophageal stenosis.^[16,17]^ This is caused by the proliferation of fibrous tissue after damage to the esophageal wall, which leads to stenosis of the esophageal lumen. The main symptoms are dysphagia, nausea, and vomiting.^[18]^ Some people may also experience sternal pain, burning sensation, and acid reflux.^[9]^ This complication may seriously affect the patient’s quality of life. Before discharge, the responsible nurse and the attending physician will develop individualized discharge education, follow-up plan and reexamination plan according to the patient’s condition. Discharge education suggested the patients should mainly eat soft food and avoid hard, hot or cold food to reduce irritation to the esophagus, maintain oral hygiene and avoid infection. Avoid swallowing too hard and try to relax the throat muscles to help food pass through the esophagus smoothly. Pay close attention to whether you have symptoms such as dysphagia. If the symptoms continue to worsen, esophagoscopy was considered to determine whether there is stenosis or other lesions in the esophagus. For mild esophageal stenosis, glucocorticoid drugs such as methylprednisolone can be considered for anti-inflammatory treatment to reduce the inflammatory response of scar tissue. In severe cases, endoscopic balloon dilatation may be required to relieve symptoms, and sometimes even multiple dilatations are required.^[19,20]^ Follow-up plan suggested that the first follow-up is 1 week after discharge. Then the follow-up was recommended every 2 weeks from 1 to 3 months after surgery and every 4 weeks from 4 to 6 months after surgery. After 7 to 12 months, the follow-up was proposed every 6 weeks and then formulate a follow-up plan based on the patient’s condition. Remind the patient to come to the hospital for a follow-up examination on time for each follow-up. Review plan: The first review is 3 weeks after discharge, and thyroid function, electrolytes, nutritional indicators, etc are examined; monthly review was suggested in patient from 1 to 3 months after surgery and every 2 months was proposed in patient from 4 to 6 months after surgery. For patients who have undergone surgery for 6 to 12 months, it is recommended to have a follow-up every 3 months, followed by a follow-up every 6 to 12 months. At the first review after discharge, the patient had normal thyroid function, good nutritional status, and no electrolyte disorder. No long-term complications of esophageal stenosis occurred during the follow-up 2 months after discharge.

### 3.4. Dispel negative emotions and return to society as soon as possible

The patient in this case was 22 years old and had a low level of psychological resilience. After the unexpected complications occurred, her mood fluctuated greatly. Based on the dual disease management theory, the nursing team of this ward developed a psychological counseling program to improve the patient’s self-efficacy.^[21,22]^ First, encourage the patient to communicate frankly with her mother, the patient was expressed with the negative emotions after the disease, and the mother express the hard work and pressure of care, so as to understand each other. Second, guide both parties to encourage each other, build empathy, express love to each other, and enhance emotional dependence. At the same time, the responsible nurse creates a comfortable treatment environment for the patient. The bed next to the window can enjoy the scenery outside the window every day to relieve psychological pressure. Because the patient fasts for a long time for enteral nutrition support, bacteria are easy to breed in the mouth, leading to bad breath, dry mouth and other discomfort symptoms. Oral care is performed for the patient every day, using saline cotton balls to scrub the mouth to keep the mouth clean, improve the patient’s comfort, and relieve negative emotions such as anxiety and fear. In addition, the responsible nurse actively communicates with the patient to understand his psychological needs, explain the disease knowledge and treatment plan to the patient, enhance the patient’s confidence in overcoming the disease, and promote the patient’s recovery.

## 4. Conclusion

Esophageal fistula is a rare and serious complication after endoscopic thyroid surgery. Early identification of postoperative complications and active causal treatment are key treatment methods. In this case, the responsible nurse closely observed the signs of postoperative esophageal fistula. After the occurrence of esophageal fistula, a multidisciplinary collaborative team was established to jointly develop treatment plans and nursing plans. We report on our nursing care experiences. By implementing progressive nutritional management, the homeostasis of the internal environment can be maintained. By developing an individualized follow-up treatment plan, including discharge education, follow-up plan, and reexamination plan, the long-term complication of esophageal stenosis can be prevented and the patient’s quality of life can be improved. Based on the actual situation of the patient, such as the patient is still young, guiding her negative emotions throughout the process, promoting his early recovery and return to society, can further improve the patient’s quality of life. Through the joint efforts of doctors, nurses and patients, it may be possible to promote the patient’s recovery and discharge from hospital.

## Author contributions

**Writing** – **original draft:** Jiamin Xu, Ying Zhou.

**Writing** – **review & editing:** Juan Lin, Huihong He.
